# Enhancing the sensitivity of the thymidine kinase assay by using DNA repair‐deficient human TK6 cells

**DOI:** 10.1002/em.22371

**Published:** 2020-04-15

**Authors:** Mahmoud Abdelghany Ibrahim, Manabu Yasui, Liton Kumar Saha, Hiroyuki Sasanuma, Masamitsu Honma, Shunichi Takeda

**Affiliations:** ^1^ Department of Radiation Genetics Kyoto University, Graduate School of Medicine Kyoto Japan; ^2^ Division of Genetics and Mutagenesis National Institute of Health Sciences Kawasaki Kanagawa Japan; ^3^ Developmental Therapeutics Branch, Center for Cancer Research National Cancer Institute, NIH Bethesda Maryland USA

**Keywords:** DNA‐damaging agent, OECD guideline, thymidine kinase assay, *TK* assay, TK6 cells

## Abstract

The OECD guidelines define the bioassays of identifying mutagenic chemicals, including the thymidine kinase (*TK*) assay, which specifically detects the mutations that inactivate the *TK* gene in the human TK6 lymphoid line. However, the sensitivity of this assay is limited because it detects mutations occurring only in the *TK* gene but not any other genes. Moreover, the limited sensitivity of the conventional *TK* assay is caused by the usage of DNA repair‐proficient *wild‐type* cells, which are capable of accurately repairing DNA damage induced by chemicals. Mutagenic chemicals produce a variety of DNA lesions, including base lesions, sugar damage, crosslinks, and strand breaks. Base damage causes point mutations and is repaired by the base excision repair (BER) and nucleotide excision repair (NER) pathways. To increase the sensitivity of *TK* assay, we simultaneously disrupted two genes encoding *XRCC1*, an important BER factor, and XPA, which is essential for NER, generating *XRCC1* ^*−/−*^ */XPA* ^*−/−*^ cells from TK6 cells. We measured the mutation frequency induced by four typical mutagenic agents, methyl methane sulfonate (MMS), cis‐diamminedichloro‐platinum(II) (cisplatin, CDDP), mitomycin‐C (MMC), and cyclophosphamide (CP) by the conventional *TK* assay using *wild‐type* TK6 cells and also by the *TK* assay using *XRCC1* ^*−/−*^ */XPA* ^*−/−*^ cells. The usage of *XRCC1* ^*−/−*^ */XPA* ^*−/−*^ cells increased the sensitivity of detecting the mutagenicity by 8.6 times for MMC, 8.5 times for CDDP, and 2.6 times for MMS in comparison with the conventional *TK* assay. In conclusion, the usage of *XRCC1* ^*−/−*^ */XPA* ^*−/−*^ cells will significantly improve *TK* assay.

## 1 INTRODUCTION

Genotoxicity assessment is essential for developing medicines and ensuring the safety of industrial chemicals. in vitro assessment of genotoxicity precedes its in vivo evaluation in the development of drugs (Corvi and Madia, 2017). The Organization for Economic Co‐operation and Development (OECD) has provided guidelines for the testing of chemicals using various in vitro genotoxicity tests including Ames test, the micronucleus test, the mouse lymphoma assay (MLA) and the thymidine kinase (*TK*) assay (TG‐471; TG‐487; TG‐490). The *TK* assay uses human lymphoblastoid TK6 cells harboring heterozygous for mutation at the *TK* gene (*TK* ^*+/−*^) and detects various mutations that inactivate the intact *TK* allelic gene including point mutations, long deletion, DNA recombination, and chromosome loss (Liber and Thilly, 1982; Koyama *et al*., 2006). The specificity of the TK assay is likely to be very high for the following reasons. The TFT selection works with extremely high specificity to kill the cells that lost the TK activity (Moore‐Brown *et al*., 1981). The TK gene is a stably expressed house‐keeping gene, and mutations of the TK gene, but not other mechanisms, cause the complete irreversible inactivation of the TK activity by the four‐hour exposure to chemicals in the TK assay (Clements, 1995). However, the sensitivity of this assay is very low especially in the detection of the chemicals that generate point mutations due to the following reasons. The size of the exons in the TK gene is 0.7 kb, which accounts for only 1.1 × 10^−7^ in the whole genome (6 × 10^6^ kb). BER normally removes over 50% of the chemical‐induced base damage within 0.5 hr (Hoch *et al*., 2017), replication forks in an asynchronous population of cells encounter only 4% (0.5/13) of the chemical‐induced base damage considering 13 hr cell cycle time of TK6, and less than 0.04% (4 × 10^−4^) of the chemical‐induced base damage causes mutations considering that the error‐rate of translesion DNA synthesis (TLS) polymerases is approximately 10^−2^ per base (McCulloch and Kunkel, 2008). Thus, chemical‐induced base damage causes mutations in the coding sequences of the TK gene with less than 4.4 × 10^−11^ probability (1.1 × 10^−7^ × 4 × 10^−4^). Approximately 20% of the missense and nonsense mutations in cancer‐related genes affect oncogenesis (Scott and Meldrum, 2005). If these mutations inactivate the TK gene also at ~20% frequency, chemical‐induced base damage inactivates the TK gene with less than 10^−11^ probability. We therefore need to damage more than 10^5^ nucleotides per cell to inactivate the TK gene at least in a single cell of ~10^6^ cells used for a TK assay. Collectively, the sensitivity of the TK assay is very low as we need to damage such a large number of nucleotides to detect the mutagenic potential of chemicals. We therefore hypothesized that the usage of DNA repair‐deficient cells might increase the sensitivity of the currently used genotoxic assays, including *TK* assay.

Chemicals induce point mutations by damaging nucleotides, inaccurate replication of such damaged nucleotides often happened during error‐prone TLS (Sale *et al*., 2012). The vast majority of damaged nucleotides are repaired by the two major pathways, base excision repair (BER) and nucleotide excision repair (NER). BER removes lesions caused by the alkylation, hydrolysis, and oxidation of nucleotides. Typical BER is initiated by an incision of the DNA strand 5′ to the damaged bases, generating single‐strand breaks (SSBs) (Krokan and Bjoras, 2013). Their repair is facilitated by the x‐ray repair cross‐complementing group 1 (XRCC1) protein, which provides docking sites for various BER effector enzymes (Thompson *et al*., 1990; Caldecott, 2003). Hypersensitivity of XRCC1 deficient cells to alkylating agents and H2O2, indicates the vital role of XRCC1 in BER (El‐Khamisy *et al*., 2003). A typical alkylating agent is MMS, which modifies both guanine (to 7‐methylguanine) and adenine (to 3‐methlyladenine) generating base mispairing and replication blocks, respectively (Beranek, 1990). NER removes helix‐destabilizing bulky adducts generated by crosslinking agents and UV (Aboussekhra *et al*., 1995). XPA recognizes such bulky adducts and is essential for initiating NER (De Vries *et al*., 1995). The capability of NER is evaluated by measuring cellular sensitivity to UV and crosslinking agents such as MMC and CDDP, which induce protein‐DNA crosslinks, intrastrand, and interstrand crosslinks (Weng *et al*., 2010; Dasari and Bernard, 2014). Both XRCC1 and XPA prevent the induction of point mutations through absolutely accurate and error‐free repair mechanisms (Lindahl, 1999). We therefore hypothesized that XRCC1/XPA double knockout cells may accumulate a higher number of mutations following a wide variety of base‐damaging substances in comparison with *wild‐type* cells.

In this study, we disrupted both XRCC1 and XPA genes in the TK6 cell line and used the resulting *XRCC1* ^*−/−*^ */XPA* ^*−/−*^ cells for the *TK* assay. We measured the mutagenicity of four mutagenic alkylating agents, MMS, CDDP, MMC, and CP following the OECD guideline for *TK* assay (TG‐490). This *TK* assay using *XRCC1* ^*−/−*^ */XPA* ^*−/−*^ cells detected 2–8 times higher numbers of mutations when compared with the conventional *TK* assay using *wild‐type* TK6 cells.

## 2 MATERIALS AND METHODS

### 2.1 Cell lines and culture conditions

The cell lines used in this study were grown in RPMI1640 medium (Gibco‐BRL, Life technology Inc., Grand Island, NY) supplemented with 10% heat‐inactivated horse serum (JRH Biosciences, Lenexa, KS), 200 μg/ml sodium pyruvate, 100 U/ml penicillin, and 100 μg/ml streptomycin, and maintained at 10^5^ to 10^6^ cells/ml at 37°C in a 5% CO_2_ atmosphere with 100% humidity (Koyama *et al*., 2006). To accurately measure spontaneously arising mutations in *TK* assay, we incubated a population of cells with CHAT (20 μM 2′‐deoxycytidine, 200 μM hypoxanthine, 0.1 μM aminopterin, T: 17.5 μM thymidine)‐containing medium for 3 days to kill *TK* ^*−/−*^ cells before starting *TK* assay (Lorge *et al*., 2016).

### 2.2 Test chemicals

We purchased MMS and CDDP from Nacalai Tesque Inc (Kyoto, Japan), MMC from Sigma‐Aldrich Inc. (CA), and CP from FUJIFILM Wako Pure Chemical Co. (Tokyo, Japan). We purchased liver S9 prepared from SD rats treated with phenobarbital and 5,6‐benzoflavone from BoZo Research Center Inc (Tokyo, Japan). All test chemicals were dissolved in phosphate‐buffered saline PBS purchased from Takara Bio Inc. (Shiga, Japan). All test chemicals were prepared immediately before the *TK* test.

### 2.3 *TK* gene mutation assay

We examined mutation frequencies as described (Koyama *et al*., 2011) and following the OECD guidelines (TG‐490). In brief, we incubated cells for 4 hr either with CDDP, MMC, or MMS in the absence of S9 mix or with CP together with S9 mix, washed with PBS, resuspended in a fresh medium and cultured for 3 days to allow for the expression of the TK deficient phenotype. To determine the plating efficiency of cells treated with mutagens, we seeded cells at 1.6 cells/well in 96‐microwell plates in the absence of TFT at day 0 (PE0) and day 3 (PE3). To count the number of cells carrying *TK* ^*−/−*^ allelic genes, we seeded the cells at day three into 96‐microwell plates at 40,000 cells/well in a medium containing 3.0 μg/ml trifluorothymidine (TFT), which kills only TK^+^ cells carrying an intact *TK* gene. All plates were incubated at 37°C in 5% CO_2_ in a humidified incubator. To determine the relative survival (RS), we scored the number of colonies in the PE3 plates at days 14 and 17 for *wild‐type* and *XRCC1* ^*−/−*^ */XPA* ^*−/−*^ TK6 cells, respectively. As the doubling time of *XRCC1* ^*−/−*^ */XPA* ^*−/−*^ was longer (16–18 hr) than that of *wild‐type* TK6 cells (11–12 hours), we found that day 17 was the suitable timing for counting surviving clones. For the scoring of mutation frequency (MF), we determined the date to count TFT‐resistant clones based on the doubling‐time of cells. We counted the number of colonies in TFT plates on day 14, then re‐supplied with TFT media, and counted again on day 28 for *wild‐type* cells. We counted the number of *XRCC1* ^*−/−*^ */XPA* ^*−/−*^ colonies on day 17, resupplied with TFT media, and counted on day 31 after plating. Mutation frequencies were calculated based on the assumption that the occurrence of mutations follows the Poisson distribution. Statistical analysis was calculated using two‐way ANOVA to analyze the statistical significance between *wild‐type* and *XRCC1* ^*−/−*^ */XPA* ^*−/−*^ TK6 cells after comparing the slopes of minimum dose responses. The statistically significant difference between spontaneously arising MFs and induced ones was calculated by the Student's *t* test. Data were generated from at least three independent experiments.

### 2.4 Disruption of *XRCC1* gene in *XPA* ^*−/−*^ TK6 cells

**FIGURE 1:**
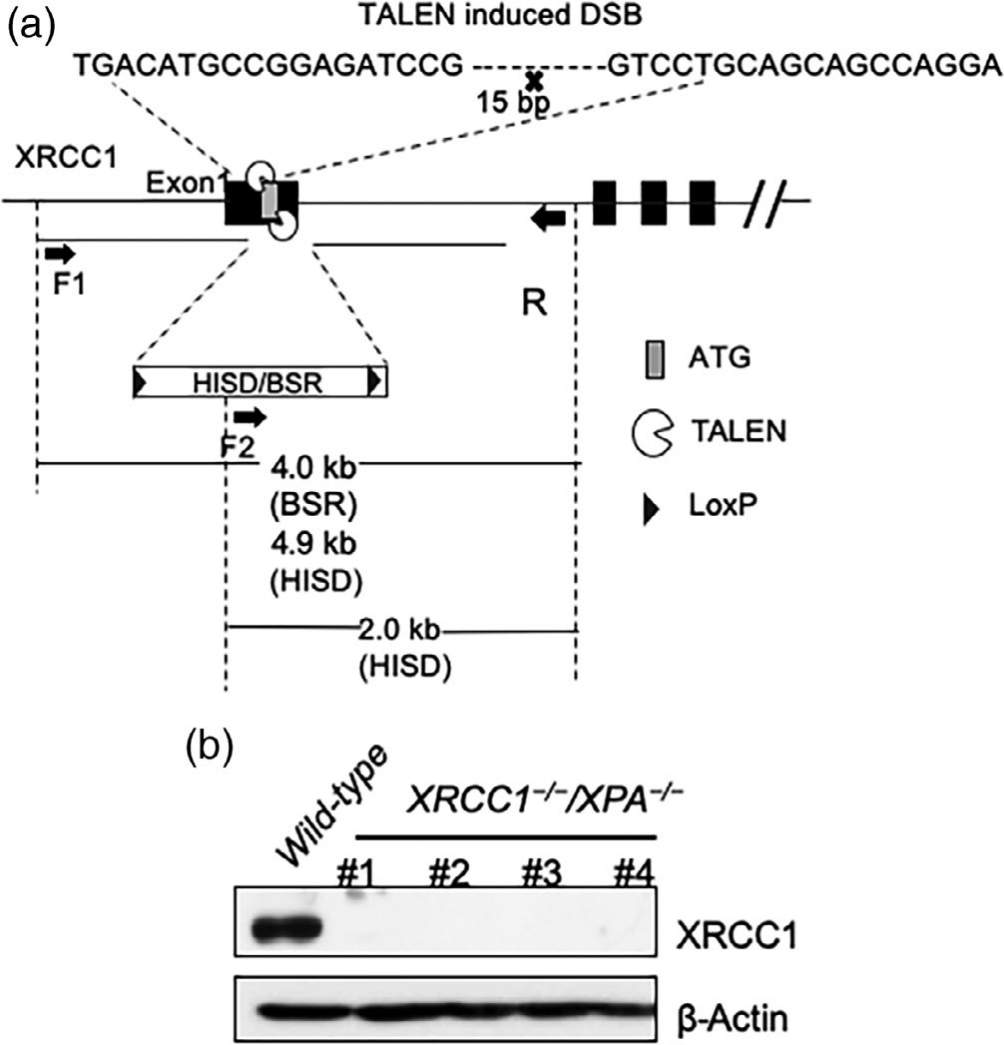
Generation of *XRCC1* ^*−/−*^ in *XPA* ^*−/−*^ TK6 cells. (a) Schematic representation of the human XRCC1 locus, base sequences of TALEN‐recognition sites, and structure of the targeting constructs containing genomic fragments and a selection‐marker gene (hisDR or bsrR) with loxP sites on both sides. The x‐mark indicates the TALEN‐induced DSB site. A pair of TALEN was designed to target exon 1. Schematic representation of the XRCC1 locus (upper) and configuration of the targeting construct carrying a marker gene (lower) flanked by ~1 kb of genomic sequence on either side. The closed boxes represent exon sequences. Note that the size of the schematic representation does not reflect the actual size of the DNA. (b) Western blot analysis of XRCC1 disrupted TK6 clones using the antibody against XRCC1 in *XPA* ^*−/−*^ human TK6 cell line

To generate *XRCC1* ^*−/−*^ */XPA* ^*−/−*^ TK6 cells, we transfected *XRCC1* targeting vector (Saha *et al*., 2018) into previously generated *XPA* ^*−/−*^ TK6 cells (Mohiuddin *et al*., 2018) after marker excision by a transiently expressed CRE‐recombinase. As we described in (Saha *et al*., 2018), we generate a pair of TALEN expression plasmids against the *XRCC1* gene, using a Golden Gate TALEN kit and a TAL effector kit (Addgene) (Cermak *et al*., 2011; Sakuma *et al*., 2013). A pair of the TALENs target sites recognizes the sequences shown in (Figure 1a), which localize at the first exon of the *XRCC1* gene. We generated the gene‐targeting constructs using DT‐A‐pA/loxP/PGK‐hisD^R^‐pA/loxP and DT‐A‐pA/loxP/PGK‐Bsr^R^‐pA/loxP vectors. Note that these vectors were generated from DT‐A‐pA/loxP/PGK‐Neo^R^‐pA/loxP (Riken Center for Life Science Technology, Japan). The genomic DNA was amplified with primers: F1 5′‐GTAGTAAAAGACAGATGCCCACAGTCCACA‐3′ and R1 5′‐GTAGTAAAAGACAGATGCCCACAGTCCACA‐3′ from the *XRCC1*‐gene locus in TK6 cells. The resulting PCR product was used as a template DNA for amplifying the 5′‐ and 3′‐arms of the gene‐targeting constructs. The 5′‐arm was amplified using the primers: F2 5′‐GCGAATTGGGTACCGGGCCGTAGTAAAAGACAGATGCC‐3′ and R2 5′‐CTGGGCTCGAGGGGGGGCCCTGGCCAGAAGGATGAGGT‐3′ of which have 15‐nt sequences identical to the upstream and downstream of the *Apa*I site in both the DT‐A‐pA/loxP/PGK‐hisD^R^‐pA/loxP and DT‐A‐pA/loxP/PGK‐Bsr^R^‐pA/loxP vectors. The 3′‐arm was amplified using the primers: F3 5′‐ TGGGAAGCTTGTCGACTTAAGACGTGAGGGAATTAATGAG‐3′ and R3 5′‐ CACTAGTAGGCGCGCCTTAAAACCACCATACCTGGCTATT‐3′, of which have 15‐nt sequences identical to the upstream and downstream of the *Afl*II site in these vectors.

The DT‐A‐pA/loxP/PGK‐hisD^R^‐pA/loxP or DT‐A‐pA/loxP/PGK‐Bsr^R^‐pA/loxP vector was digested with both *Apa*I and *Afl*II, which cut at the 5′ and 3′ of the selection marker genes, respectively. We combined the digested DNAs with the 5′‐ and 3′‐arms using the Seamless Cloning and Assembly Kit (Thermo Fisher Scientific). We transfected 6 μg each of the TALEN‐expression plasmids and 2 μg each of the two gene‐targeting vectors carrying *hisD* and *Bsr* ^*R*^ into 4 × 10^6^ TK6 cells using the Neon Transfection System (Life Technologies) with three times 1,350 V pulse with a 10 ms pulse width. After electroporation, we released cells into a 20 ml drug‐free medium containing 10% horse serum and incubated them for 48 hr. We seeded cells into 96‐well plates with both histidinol and blasticidin S antibiotics and incubated for 2 weeks. We confirmed the loss of XRCC1 protein expression in individual clones by western blot analysis (Figure 1b).

### 2.5 Generation of *XRCC1* ^*−/−*^ */XPA* ^*−/−*^ heterozygous for the *TK* gene for *TK* assay

We disrupted the *XRCC1* gene in *XPA* ^*−/−*^ TK6 cells derived from a TSCER2 subline carrying compound heterozygous mutations of the *TK* allelic gene (*TK* ^*−/−*^) (Honma *et al*., 2007). Cells (5 × 10^6^) of *XRCC1* ^*−/−*^ */XPA* ^*−/−*^ TSCER2 (*TK* ^*−/−*^) were suspended in 0.1 ml Nucleofector Solution V and were cotransfected with 50 μg of pCBASce vector and 2 μg of targeting vector pTK15 plasmid (Honma *et al*., 2003) using Nucleofector I according to the manufacturer's recommendations (Maasho *et al*., 2004) Subsequently, the cells were cultured for 72 hr and were then seeded into 96‐microwell plates in the presence of HAT (200 μM hypoxanthine, 0.1 μM aminopterin, and 17.5 μM thymidine) to isolate TK+/− revertant TSCER2 clones. The drug‐resistant colonies were isolated 2 weeks later and were independently cultured for DNA analysis.

### 2.6 Western blot

Cells (1 × 10^6^) were lysed in 100 μl sodium dodecyl sulfate (SDS) buffer, containing Tris–HCl (25 mM, pH 6.5), SDS (1%), β‐mercaptoethanol (0.24 mM), bromophenol blue (0.1%), and glycerol (5%). Whole‐cell extracts were separated by electrophoresis, transferred onto polyvinylidene difluoride membranes, and blocked in 5% skimmed milk dissolved in Tween‐20 (0.1%) in TBS. Membranes were incubated with primary antibodies (Rabbit polyclonal α‐XPA, Santa Cruz) overnight at 4°C, followed by washing in Tween‐20 (0.1%) in TBS. Membranes were incubated with appropriate HRP‐linked secondary antibodies (Goat polyclonal α‐rabbit HRP, Santa Cruz) at room temperature for 1 hr and washed thrice before signal detection. Membranes were developed by chemiluminescence using ECL reagent.

## 3 RESULTS

### 3.1 The *TK* assay using *XRCC1* ^*−/−*^ */XPA* ^*−/−*^ TK6 cells detected a higher CP‐MF response without a significant increase in the TK assay sensitivity

**FIGURE 2:**
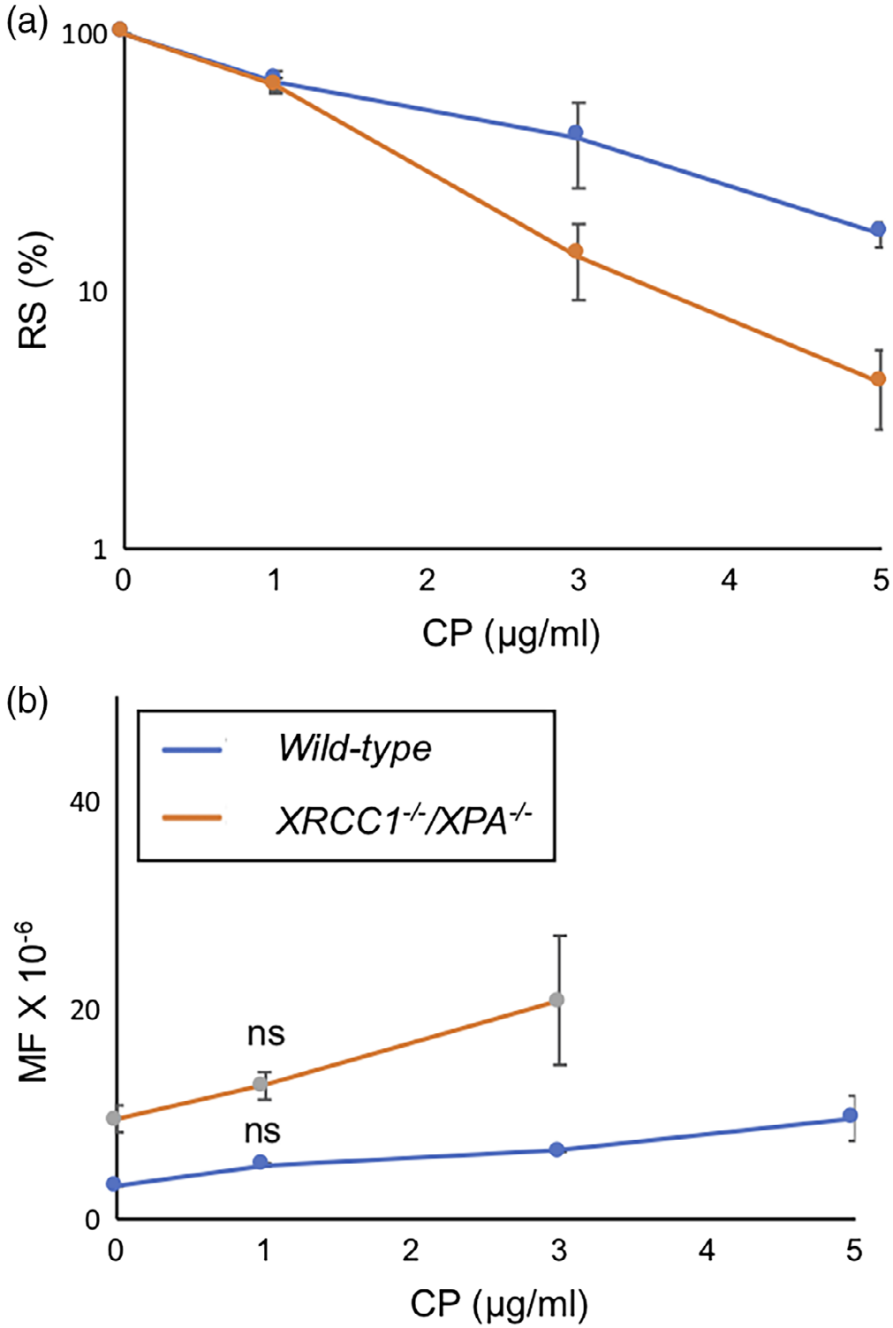
The mutation frequency (MF) of *wild‐type* and *XRCC1* ^*−/−*^ */XPA* ^*−/−*^ TK6 cells induced by CP. The percentage survival of cells following exposure of cells to the indicated concentrations of CP (a). Hundred percentage is the survival of untreated cells in (a). The average MF of cells to the indicated concentrations of CP (b). Error bars represent *SD* from at least three independent experiments. The statistically significant difference between spontaneously arising MFs and induced ones was calculated by the Student's *t* test. We defined *p*‐value < .05 as statistically significant and mark such difference with *. (ns) *p*‐value was not significant

To determine if the usage of *XRCC1* ^*−/−*^ */XPA* ^*−/−*^ cells enhances the sensitivity of the *TK* assay, we firstly examined the mutagenicity of CP in the presence of S9 mix. The mutation frequencies of *wild‐type* and *XRCC1* ^*−/−*^ */XPA* ^*−/−*^ TK6 cells linearly increased with the dose of CP (Figure 2b). Since the highest dose (5 μg/ml) killed over 95% of the *XRCC1* ^*−/−*^ */XPA* ^*−/−*^ mutant (Figure 2a), we chose 3 μg/ml CP as of the highest dose for *XRCC1* ^*−/−*^ */XPA* ^*−/−*^. The average frequency of spontaneously arising mutation (hereafter called spontaneous MF) of *wild‐type* TK6 cells was 4.1 ± 1.2 × 10^−6^ and their MF was 5.7 ± 0.7 × 10^−6^ at 1 μg/ml CP and 6.6 ± 0.8 × 10^−6^ at 3 μg/ml CP (Figure 2b). Spontaneous MF of *XRCC1* ^*−/−*^ */XPA* ^*−/−*^cells was 9.5 ± 1.3 × 10^−6^, and their MF was 12.7 ± 1.3 × 10^−6^ at 1 μg/ml CP and 20.9 ± 6.1 × 10^−6^ at 3 μg/ml CP (Figure 2b). Collectively, after comparing the slopes of the minimum dose–response, we found that the usage of *XRCC1* ^*−/−*^ */XPA* ^*−/−*^ cells for the TK assay increases the detection of the mutagenicity associated with 1 and 3 μg/ml CP by 4.5 folds in comparison with the conventional *TK* assay, *p*‐value = .02 (Table S1). However, the difference between spontaneously arising MFs and induced ones at the minimum concentration of CP was not statistically significant in *wild‐type* or *XRCC1* ^*−/−*^ */XPA* ^*−/−*^cells (Figure 2b).

### 3.2 The usage of *XRCC1* ^*−/−*^ */XPA* ^*−/−*^ TK6 cells allows for detecting a ~ three times higher number of MMS‐induced mutations than the conventional *TK* assay

We tested the new TK assay using *XRCC1* ^*−/−*^ */XPA* ^*−/−*^ TK6 cells for detecting mutagenicity of MMC, CDDP, and MMS without adding (S9 mix) (Honma *et al*., 1997) as they did not require exogenous metabolic activation to induce their mutagenicity in our experiments. This protocol was in agreement with the latest recommendations proposed by the international workshop for genotoxicity testing (IWGT) (Gollapudi *et al*., 2019). The spontaneous MF was 7.0 ± 1.4 × 10^−6^ for *wild‐type* and 13.3 ± 1.1 × 10^−6^ for *XRCC1* ^*−/−*^ */XPA* ^*−/−*^ TK6 cells without S9 mix, in which MFs were ~ 2 times higher than the spontaneous MF with S9 mix. The higher spontaneous MFs might be due to the usage of different batch of horse serum since spontaneous MF is not supposed to be affected by the absence or presence of S9 mix as previously described (Koyama *et al*., 2006).

**FIGURE 3:**
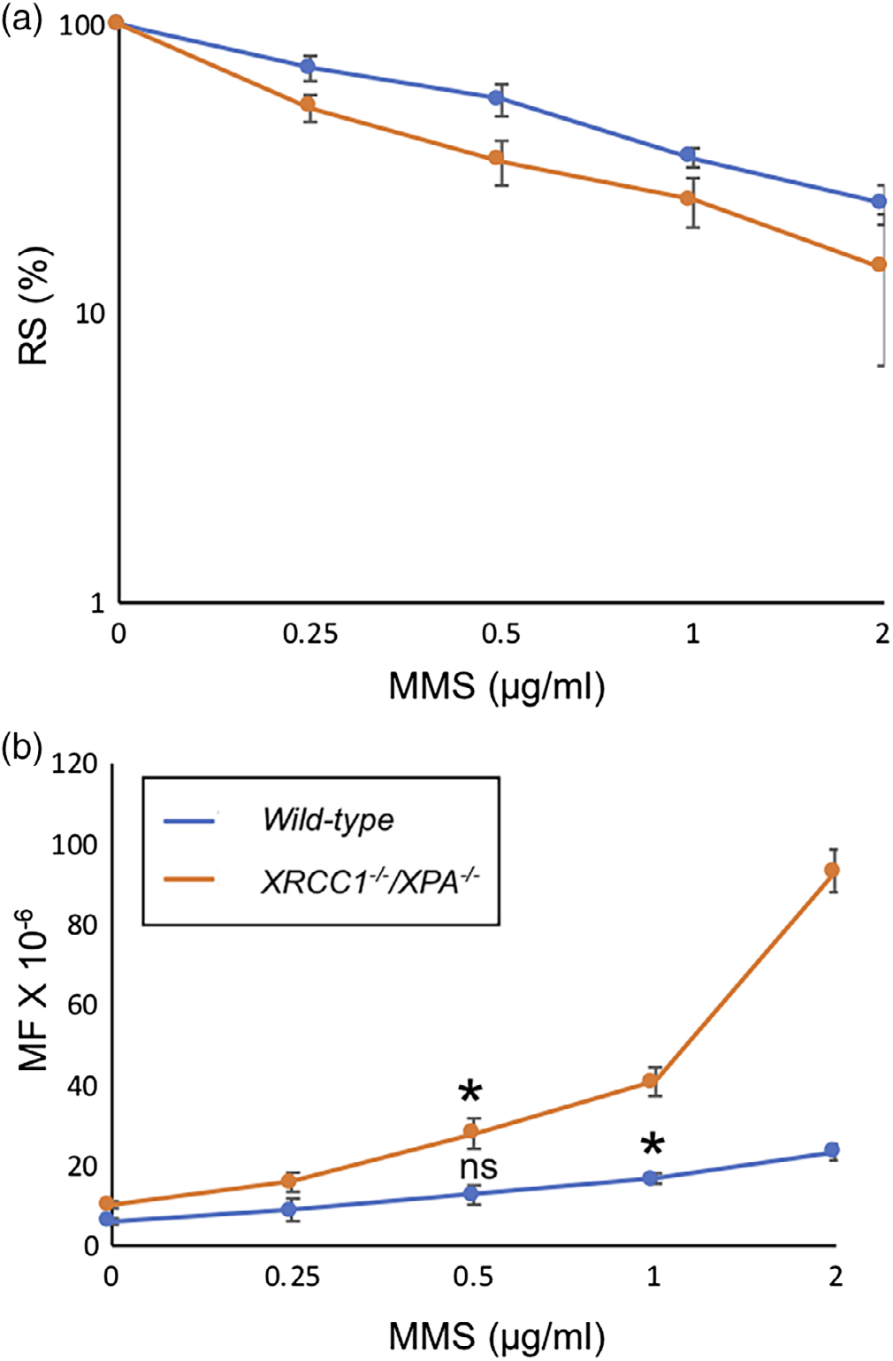
The mutation frequency (MF) of *wild‐type* and *XRCC1* ^*−/−*^ */XPA* ^*−/−*^ TK6 cells induced by MMS. Data are shown as in Figure 2. Error bars represent *SD* from at least three independent experiments. The statistically significant difference between spontaneously arising MFs and induced ones was calculated by the Student's *t* test. We defined *p*‐value < .05 as statistically significant and mark such difference with *. (ns) *p*‐value was not significant

We measured MMS‐induced mutagenesis comparing the new *TK* assay using *XRCC1* ^*−/−*^ */XPA* ^*−/−*^ TK6 cells with the conventional *TK* assay. The relative survival (RS) was comparable between *wild‐type* and *XRCC1* ^*−/−*^ */XPA* ^*−/−*^ TK6 cells; 70% for *wild‐type* and 50% for *XRCC1* ^*−/−*^ */XPA* ^*−/−*^at 0.25 μg/ml MMS (Figure 3a). The MF for *wild‐type* TK6 cells was 8.9 ± 2.8 × 10^−6^ at 0.25 μg/ml MMS and 12.6 ± 2.4 × 10^−6^ at 0.5 μg/ml MMS (Figure 3b). The MF of *XRCC1* ^*−/−*^ */XPA* ^*−/−*^ was 15.8 ± 2.3 × 10^−6^ at 0.25 μg/ml MMS and 27.9 ± 3.7 × 10^−6^ at 0.5 μg/ml MMS (Figure 3b). Comparing the slopes of the dose–response showed that the usage of *XRCC1* ^*−/−*^ */XPA* ^*−/−*^cells for the TK assay increases the sensitivity of detecting the mutagenicity associated with 0.25 and 0.5 μg/ml MMS by 2.6 folds in comparison with the conventional *TK* assay, *p*‐value <.0001 (Table S2). To determine the minimum concentrations of MMS that induced mutations in a statistically significant manner, we compared the difference between spontaneously arising MFs and induced ones. *XRCC1* ^*−/−*^ */XPA* ^*−/−*^ TK6 shows a significant detection of mutations at 0.25 μg/ml MMS (*p*‐value = .036), while *wild‐type* TK6 shows a significant induction of mutations at 0.5 μg/ml MMS (*p*‐value = .02) but not at 0.25 μg/ml MMS (*p*‐value = .23) (Figure 3b).

### 3.3 *TK* assay using *XRCC1* ^*−/−*^ */XPA* ^*−/−*^ TK6 cells detected several times higher number of mutations induced by crosslinking agents, MMC and CDDP, than the conventional *TK* assay

**FIGURE 4:**
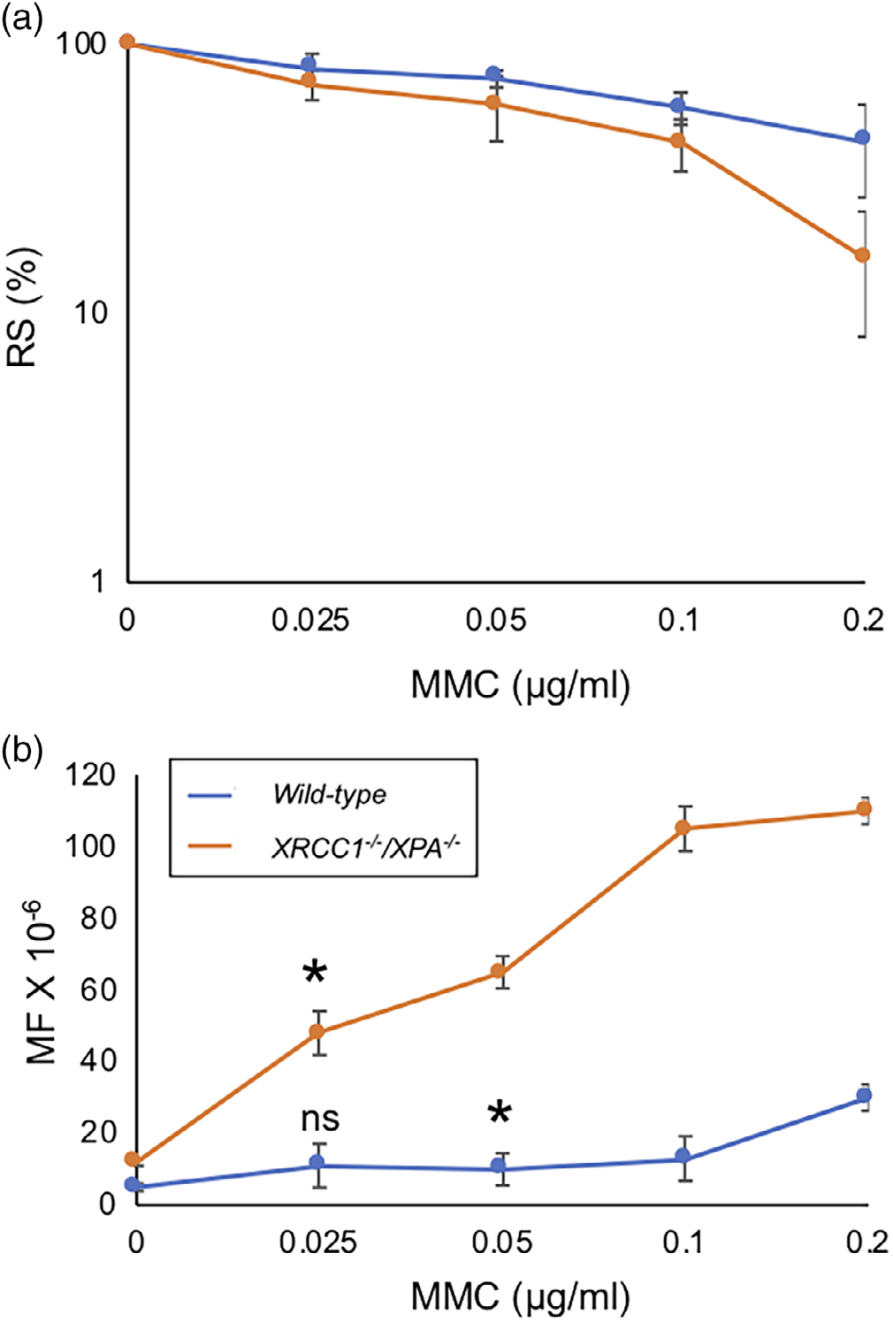
The mutation frequency (MF) of *wild‐type* and *XRCC1* ^*−/−*^ */XPA* ^*−/−*^ TK6 cells induced by MMC. Data are shown as in Figure 2. Error bars represent *SD* from at least three independent experiments. The statistically significant difference between spontaneously arising MFs and induced ones was calculated by the Student's *t* test. We defined *p*‐value < .05 as statistically significant and mark such difference with *. (ns) *p*‐value was not significant

The RS was comparable between *wild‐type* and *XRCC1* ^*−/−*^ */XPA* ^*−/−*^ TK6 cells; 80% at 0.025 μg/ml MMC for *wild‐type* and 70% at 0.025 μg/ml MMC for *XRCC1* ^*−/−*^ */XPA* ^*−/−*^ (Figure 4a). The MF of *wild‐type* was 11.4 ± 1.1 × 10^−6^ at 0.025 μg/ml MMC and 13 ± 1.1 × 10^−6^ at 0.05 μg/ml MMC (Figure 4b). The MF of *XRCC1* ^*−/−*^ */XPA* ^*−/−*^ was 48 ± 6.1 × 10^−6^ at 0.025 μg/ml MMC and 65 ± 4.5 × 10^−6^ at 0.05 μg/ml MMC. The usage of *XRCC1* ^*−/−*^ */XPA* ^*−/−*^cells increases MF response by 8.6 folds after comparing the slopes of the dose–response at 0.025, 0.05 μg/ml MMC with *wild‐type* TK6 cell line, *p*‐value <.0001 (Table S3). After comparing the difference between spontaneously arising MFs and induced ones, XRCC*1* ^*−/−*^ */XPA* ^*−/−*^ TK6 shows a significant detection of mutations at 0.025 μg/ml MMC (*p*‐value = .001), while *wild‐type* TK6 shows a significant induction of mutations at 0.05 μg/ml MMC (*p*‐value = .030) but not at 0.025 μg/ml MMC (*p*‐value = .063) (Figure 4b).

**FIGURE 5:**
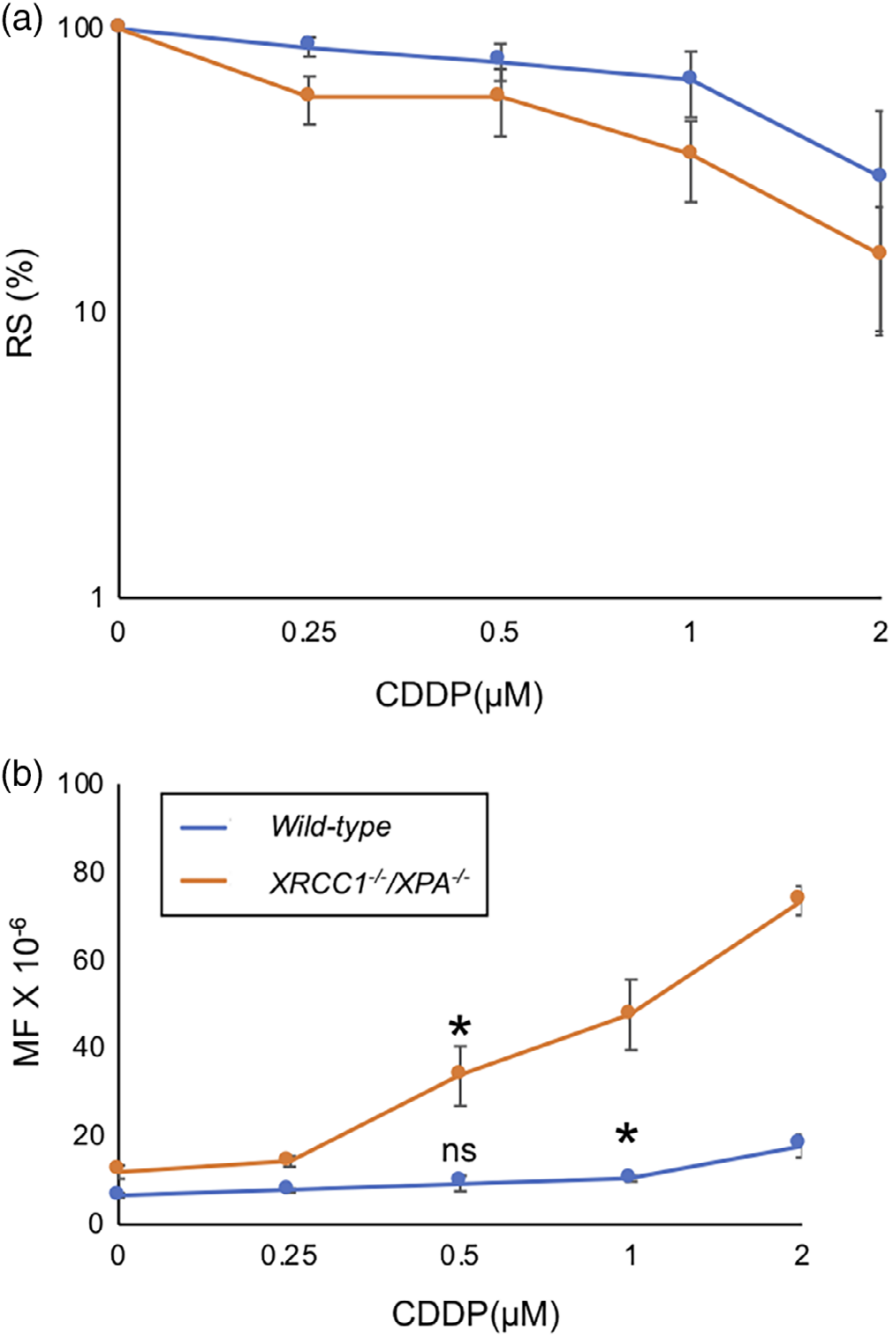
The mutation frequency (MF) of *wild‐type* and *XRCC1* ^*−/−*^ */XPA* ^*−/−*^ TK6 cells induced by CDDP. Data are shown as in Figure 2. Error bars represent *SD* from at least three independent experiments. The statistically significant difference between spontaneously arising MFs and induced ones was calculated by the Student's *t* test. We defined *p*‐value < .05 as statistically significant and mark such difference with *. (ns) *p*‐value was not significant

We then examined CDDP‐induced mutagenicity at 0.25 μM and 0.5 μM CDDP, which decreased RS by less than 50% in both genotypes (Figure 5a). The MF for *wild‐type* was 7.7 ± 0.4 × 10^−6^ at 0.25 μM CDDP and 9.3 ± 1.7 × 10^−6^ at 0.5 μM CDDP (Figure 5b). The MF of *XRCC1* ^*−/−*^ */XPA* ^*−/−*^ was 14.3 ± 1.1 × 10^−6^ at 0.25 μM CDDP and 33 ± 6.7 × 10^−6^ at 0.5 μM CDDP (Figure 5b). RS was more than 50% (Figure 5a). The dose–response slope for *XRCC1* ^*−/−*^ */XPA* ^*−/−*^ showed 8.5 fold increase than *wild‐type* TK6 at 0.25, 0.5 μM CDDP, *p*‐value <.0001 (Table S4). After comparing the difference between spontaneously arising MFs and induced ones, *XRCC1* ^*−/−*^ */XPA* ^*−/−*^ TK6 detected a significant detection of mutations at 0.5 μM CDDP (*p*‐value = .039), while the TK assay using *wild‐type* TK6 detected a significant induction of mutations at 1.0 μM CDDP (*p*‐value = .002) but not at 0.5 μM CDDP (*p*‐value = .106) (Figure 5b).

## 4 DISCUSSION

In the present study, we showed that the usage of *XRCC1* ^*−/−*^ */XPA* ^*−/−*^ cells for the *TK* assay increased its sensitivity in detecting the mutagenicity of various DNA cross‐linking and alkylating agents. Using this new *TK* assay, we studied four typical mutagens, CP, MMS, MMC, and CDDP. The usage of *XRCC1* ^*−/−*^ */XPA* ^*−/−*^ cells gives a higher number of MF response by 4.5 times for CP, 2.6 times for MMS, 8.6 times for MMC, and 8.5 times for CDDP in comparison with the conventional *TK* assay.

To check the ability of *XRCC1* ^*−/−*^ */XPA* ^*−/−*^ cells to detect weak mutagens with more sensitivity, we determine the minimum doses of the genotoxic agents whose doses increased the MF in a statistically significant manner. We found that the TK assay using *XRCC1* ^*−/−*^ */XPA* ^*−/−*^ TK6 enhanced a significant detection of mutations at the minimum concentrations of DNA cross‐linking agents MMC (0.025 μg/ml) and CDDP (0.5 μM) while the *wild‐type* TK6 could not detect similar significant increase (Figures 4b and 5b). Similarly, the TK assay using *XRCC1* ^*−/−*^ */XPA* ^*−/−*^ TK6 detected a significant number of mutations at the minimum concentration of DNA alkylating agent MMS (0.5 μg/ml) while the *wild‐type* TK6 could not detect similar significant increase (Figure 3b). We could not detect a similar enhanced sensitivity for detecting CP because the slope of dose‐MF was similar between *wild‐type* and *XRCC1* ^*−/−*^ */XPA* ^*−/−*^ cells (Figure 2b). CP generates DNA cleavage, crosslinks, and adducts (reviewed in Ozolinsc̆, 2010), and MMS is a mono‐alkylating agent (Sobol *et al*., 2002). MMC and CDDP, on the other hand, are DNA cross‐linking agents generating a variety of lesions, including protein‐DNA crosslinks, intrastrand crosslinks, and interstrand crosslinks (Tomasz, 1995; Jordan and Carmo‐Fonseca, 2000; Lorenti Garcia *et al*., 2009). XPA and XRCC1 participate in the repair of crosslinks, and these pathways have an overlapping role in removing a fraction of the crosslink DNA lesions (Zheng *et al*., 2003; Mustra *et al*., 2007; Zhang and Walter, 2014; Semlow *et al*., 2016). This overlapping role may explain why the usage of *XRCC1* ^*−/−*^ */XPA* ^*−/−*^ cells increased the sensitivity of the *TK* assay for detecting the mutagenicity of MMC and CDDP to greater extents when compared with MMS, which induces the DNA lesions that are repaired exclusively by XRCC1‐dependent BER (Op Het Veld *et al*., 1998). In summary, the usage of *XRCC1* ^*−/−*^ */XPA* ^*−/−*^ cells is advantageous, particularly when the *TK* assay examines the mutagenicity of crosslinking agents.

Enhancing the performance of the in vitro genotoxicity testing would lead to more reliance on the in vitro tests and less of a need to use in vivo tests. The EURL EVCAM has requested the improvement of the individual in vitro genotoxicity detection assays to increase their overall performance and as a consequence, minimize or even prevent the use of animals for detecting mutagenic chemicals (Corvi and Madia, 2017; EURL ECVAM, 2013a). A significant concern of the mammalian cell‐based gene mutation tests is their limited sensitivity, and these tests need very high concentrations of chemicals, whose genotoxicity might not be extrapolated to the genotoxicity of environmentally appropriate lower levels of the substances (Elespuru *et al*., 2009). The usage of DNA repair‐deficient cells may solve this problem by increasing the sensitivity of the metazoan cell‐based gene mutation tests (Ji *et al*., 2009; Yamamoto *et al*., 2011; Nishihara *et al*., 2016). Moreover, this usage gives an insight into molecular mechanisms underlying the mutagenicity of chemicals. For example, higher induced MF in *XRCC1* ^*−/−*^ */XPA* ^*−/−*^ cells than *wild‐type* cells indicate that relevant compounds cause the DNA lesions that are repaired by either XRCC1‐dependent BER or XPA‐dependent NER. We propose the following two‐step examination of genotoxic chemicals; first, the identification of a wide variety of genotoxic chemicals using a few mutants such as *XRCC1* ^*−/−*^ */XPA* ^*−/−*^ and double‐strand‐break repair mutant cells (Hsieh *et al*., 2019), and subsequently, characterization of molecular mechanisms underlying the identified genotoxicity using cells deficient in individual repair pathways that repair specific lesions. Our proposal for the multistep process is not for routinely evaluating genotoxicity but for investigating mutagenic mechanisms for special purposes, for example, the elucidation of molecular mechanisms underlying the mutagenesis of different chemicals in academic research. In conclusion, we propose that the usage of *XRCC1* ^*−/−*^ */XPA* ^*−/−*^ cells will improve the sensitivity of the *TK* assay; however, further validation is still needed before requesting it for regulatory use.

## AUTHOR CONTRIBUTIONS

Shunichi Takeda and Mahmoud Abdelghany Ibrahim designed the basic framework of this study. Mahmoud Abdelghany Ibrahim performed all experiments. Mahmoud Abdelghany Ibrahim prepared the manuscript draft with intellectual input from Shunichi Takeda, Masamitsu Honma, Manabu Yasui, and Shunichi Takeda finalized the manuscript. Manabu Yasui, Liton Kumar Saha provided information for designing some of the experiments. Masamitsu Honma provided isogenic TK6 *wild‐type* cells. Hiroyuki Sasanuma and Manabu Yasui advised Mahmoud Abdelghany Ibrahim about technical issues during the experimentation. All authors have approved the final manuscript.

## Supporting information


**Appendix** **S1:** Supplementary Inforamtion
