# Supplementary material for: Enhancing the sensitivity of the thymidine kinase assay by using DNA repair‐deficient human TK6 cells
Source: Environ Mol Mutagen. 2020 Apr 15;61(6):602–10. doi: 10.1002/em.22371 (PMC7384079; doi:10.1002/em.22371)
Supplement: Supplementary file 1 — Appendix S1: Supplementary Inforamtion [file EM-61-602-s001.docx]

| CP Concentration μg/ml | *wild-type* MF | *XRCC1^−/−^/XPA^−/−^* MF |
| --- | --- | --- |
| 0 | 4.1 ± 1.2 × 10^-6^ | 9.5 ± 1.3 × 10^-6^ |
| 1 | 5.7 ± 0.7 × 10^-6^ | 12.7 ± 1.3 × 10^-6^ |
| 3 | 6.6 ± 0.8 × 10^-6^ | 20.9 ± 6.1 × 10^-6^ |

Table S1

| MMS Concentration μg/ml | *wild-type* MF | *XRCC1^−/−^/XPA^−/−^* MF |
| --- | --- | --- |
| 0 | 7.0 ± 1.4 × 10^-6^ | 13.3 ± 1.1 × 10^-6^ |
| 0.25 | 8.9 ± 2.8 × 10^-6^ | 15.8 ± 2.3 × 10^-6^ |
| 0.5 | 12.6 ± 2.4 × 10^-6^ | 27.9 ± 3.7 × 10^-6^ |

| MMC Concentration μg/ml | *wild-type* MF | *XRCC1^−/−^/XPA^−/−^* MF |
| --- | --- | --- |
| 0 | 7.0 ± 1.4 × 10^-6^ | 13.3 ± 1.1 × 10^-6^ |
| 0.025 | 11.4 ± 1.1 × 10^-6^ | 48 ± 6.1 × 10^-6^ |
| 0.05 | 13 ± 1.1 × 10^-6^ | 65 ± 4.5 × 10^-6^ |

| CDDP Concentration μM | *wild-type* MF | *XRCC1^−/−^/XPA^−/−^* MF |
| --- | --- | --- |
| 0 | 7.0 ± 1.4 × 10^-6^ | 13.3 ± 1.1 × 10^-6^ |
| 0.25 | 7.7 ± 0.4 × 10^-6^ | 14.3 ± 1.1 × 10^-6^ |
| 0.5 | 9.3 ± 1.7 × 10^-6^ | 33 ± 6.7 × 10^-6^ |

*XRCC1^−/−^/XPA^−−^* CDDP induced MF is 8.5 folds more than *wild-type* cells. *p*-value < 0.0001

*XRCC1^−/−^/XPA^−/−^* MMC induced MF is 8.6 folds more than *wild-type* cells. *p*-value < 0.0001

*XRCC1^−/−^/XPA^−/−^* MMS induced MF is 2.6 folds more than *wild-type* cells. *p*-value < 0.0001

*XRCC1^−/−^/XPA^−/−^* CP induced MF is 4.5 folds more than *wild-type*. *p*-value = 0.02

Table S2

S

Table S3

S

Table S4

Tables S1-S4, Showed the actual CP, MMS, MMC, CDDP doses range used for comparing the slopes of the induced mutation frequencies (MF) between Wild type and *XRCC1^−/−^/XPA^−/−^* TK6 cells. Induced MF comparisons were calculated following the formula:

*XRCC1^−/−^/XPA^−/−^* (B – A)

*Wild-type* (B – A)

Where B= MF at the highest dose in each table, A= MF without treatment of DNA damaging agent. Statistical significance was computed using two-way ANOVA.
